# Efficacy of levosimendan infusion in patients undergoing a left ventricular assist device implant in a propensity score matched analysis of the EUROMACS registry—the Euro LEVO-LVAD study

**DOI:** 10.1093/ejcts/ezad095

**Published:** 2023-03-13

**Authors:** Mahmoud Abdelshafy, Kadir Caliskan, Andrew J Simpkin, Ahmed Elkoumy, Jesse R Kimman, Hagar Elsherbini, Hesham Elzomor, Theo M M H de By, Can Gollmann-Tepeköylü, Michael Berchtold-Herz, Antonio Loforte, David Reineke, Felix Schoenrath, Lech Paluszkiewicz, Jan Gummert, Paul Mohacsi, Bart Meyns, Osama Soliman

**Affiliations:** Discipline of Cardiology, Saolta Healthcare Group, Galway University Hospital, Health Service Executive, Galway, Ireland; CORRIB Core Lab, University of Galway, Galway, Ireland; Department of Cardiology, Al-Azhar University, Cairo, Egypt; Department of Cardiology, Erasmus MC University Medical Center, Rotterdam, Netherlands; School of Mathematical and Statistical Sciences, University of Galway, Galway, Ireland; Insight Centre for Data Analytics, University of Galway, Galway, Ireland; Discipline of Cardiology, Saolta Healthcare Group, Galway University Hospital, Health Service Executive, Galway, Ireland; CORRIB Core Lab, University of Galway, Galway, Ireland; Islamic Center of Cardiology and Cardiac Surgery, Al-Azhar University, Cairo, Egypt; Department of Cardiology, Erasmus MC University Medical Center, Rotterdam, Netherlands; Department of Intensive Care, Erasmus MC University Medical Center, Rotterdam, Netherlands; Department of Cardiology, Erasmus MC University Medical Center, Rotterdam, Netherlands; Discipline of Cardiology, Saolta Healthcare Group, Galway University Hospital, Health Service Executive, Galway, Ireland; CORRIB Core Lab, University of Galway, Galway, Ireland; Islamic Center of Cardiology and Cardiac Surgery, Al-Azhar University, Cairo, Egypt; EACTS House, Windsor, United Kingdom; Department of Cardiac Surgery, Medical University of Innsbruck, Innsbruck, Austria; Department of Cardiovascular Surgery, Faculty of Medicine, Heart Center Freiburg University, University of Freiburg, Freiburg, Germany; Division of Cardiac Surgery, S. Orsola University Hospital, ALMA Mater Studiorum University of Bologna, IRCCS Bologna, Bologna, Italy; Department of Surgical Sciences, University of Turin, Turin, Italy; Department of Cardiovascular Surgery, University Hospital, Berne, Switzerland; Department of Cardiothoracic and Vascular Surgery, German Heart Center Berlin, Berlin, Germany; DZHK (German Centre for Cardiovascular Research), Partner Site, Berlin, Germany; Department for Thoracic and Cardiovascular Surgery, Heart and Diabetes Centre NRW, Ruhr-University Bochum, Bad Oeynhausen, Germany; Department for Thoracic and Cardiovascular Surgery, Heart and Diabetes Centre NRW, Ruhr-University Bochum, Bad Oeynhausen, Germany; HerzGefässZentrum im Park, Zürich, Switzerland; Department of Internal Medicine, Division of Cardiology, Medical University of Graz, Graz, Austria; Katholieke Universiteit Leuven, Leuven, Belgium; Discipline of Cardiology, Saolta Healthcare Group, Galway University Hospital, Health Service Executive, Galway, Ireland; CORRIB Core Lab, University of Galway, Galway, Ireland; CÚRAM Centre for Medical Devices, Galway, Ireland

**Keywords:** levosimendan, LVAD, Right-sided heart failure, propensity score matching, mechanical circularity support, heart failure

## Abstract

**OBJECTIVES:**

Early right-sided heart failure (RHF) was seen in 22% of recipients of a left ventricular assist device (LVAD) in the European Registry for Patients with Mechanical Circulatory Support (EUROMACS). However, the optimal treatment of post-LVAD RHF is not well known. Levosimendan has proven to be effective in patients with cardiogenic shock and in those with end-stage heart failure. We sought to evaluate the efficacy of levosimendan on post-LVAD RHF and 30-day and 1-year mortality.

**METHODS:**

The EUROMACS Registry was used to identify adults with mainstream continuous-flow LVAD implants who were treated with preoperative levosimendan compared to a propensity matched control cohort.

**RESULTS:**

In total, 3661 patients received mainstream LVAD, of which 399 (11%) were treated with levosimendan pre-LVAD. Patients given levosimendan had a higher EUROMACS RHF score [4 (2– 5.5) vs 2 (2– 4); *P* < 0.001], received more right ventricular assist devices (RVAD) [32 (8%) vs 178 (5.5%); *P* = 0.038] and stayed longer in the intensive care unit post-LVAD implant [19 (8–35) vs 11(5–25); *P* < 0.001]. Yet, there was no significant difference in the rate of RHF, 30-day, or 1-year mortality. Also, in the matched cohort (357 patients taking levosimendan compared to an average of 622 controls across 20 imputations), we found no evidence for a difference in postoperative severe RHF, RVAD implant rate, length of stay in the intensive care unit or 30-day and 1-year mortality.

**CONCLUSIONS:**

In this analysis of the EUROMACS registry, we found no evidence for an association between levosimendan and early RHF or death, albeit patients taking levosimendan had much higher risk profiles. For a definitive conclusion, a multicentre, randomized study is warranted.

**Figure:**
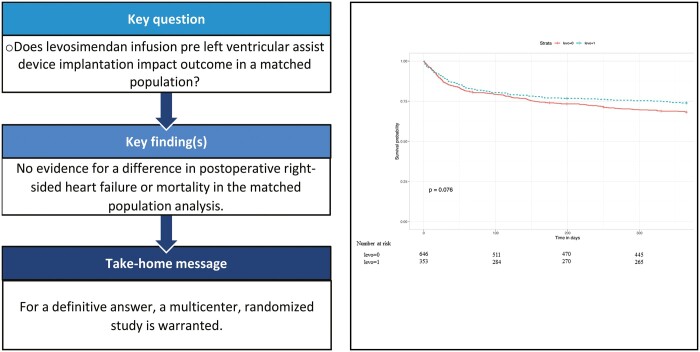

## INTRODUCTION

Right-sided heart failure (RHF) following left ventricular assist device (LVAD) surgery has been reported in 4% to 50% of patients [1–6]. In the European Registry for Patients with Mechanical Circulatory Support (EUROMACS) RHF risk score study, early severe RHF was seen in more than a fifth of patients, resulting in 37%, 39% and 47% deaths at 3, 6 and 12 months, respectively [6].

In contrast to conventional inotropes, levosimendan is a unique inotropic agent that acts as a calcium sensitizer. It sensitizes troponin C without increasing intracellular calcium concentration or exacerbating ischaemia and acts as an inodilator, thereby reducing the cardiac pre- and afterload. Furthermore, levosimendan acts as a potassium channel opener; it has an active metabolite (OR1896) that peaks approximately 80 to 90 h after administration and is associated with haemodynamic improvements that are sustained for a week [7]. The advantages of levosimendan include beneficial symptomatic, haemodynamic and neurohormonal effects and improved peripheral organ perfusion and renal function. Importantly, there is no effect attenuation in patients using a beta-blocker [8], which is one of the main heart failure (HF) treatment agents. In 2 recent meta-analyses, levosimendan use was associated with improvement of a wide range of haemodynamic and clinical outcomes in patients undergoing extracorporeal membrane oxygenation (ECMO) support [9] and in patients with end-stage HF [10]. However, despite more than a decade long use of LVAD and 2 decades of the use of levosimendan in patients with HF, the literature on levosimendan use in patients with an LVAD is limited [11]. We used the EUROMACS database to evaluate the effectiveness of levosimendan on the occurrence of postoperative RHF, the need for an RVAD implant, lengths of stays in the intensive care unit (ICU), and 30-day and 1-year deaths in patients undergoing LVAD implants compared to propensity score-matched controls.

## METHODS

### Ethical approval

This study was approved by the EUROMACS scientific review committee on 28 June 2021 (EUROMACS study number 58). Participation in the EUROMACS Registry was approved by the institutional review committees of all respective participating centres, and all subjects gave informed consent. Furthermore, this study complied with the principles outlined in the Declaration of Helsinki.

### Study design

EUROMACS is a registry of the European Association for Cardio-Thoracic Surgery. In this registry, all relevant clinical, echocardiographic, haemodynamic and laboratory parameters of patients who received mechanical circulatory support have been collected prospectively since January 2011. Detailed descriptions of the database and the collection procedure were provided previously [12]. The manuscript was composed according to the STROBE guidelines [13].

### Patients

**Figure 1:**
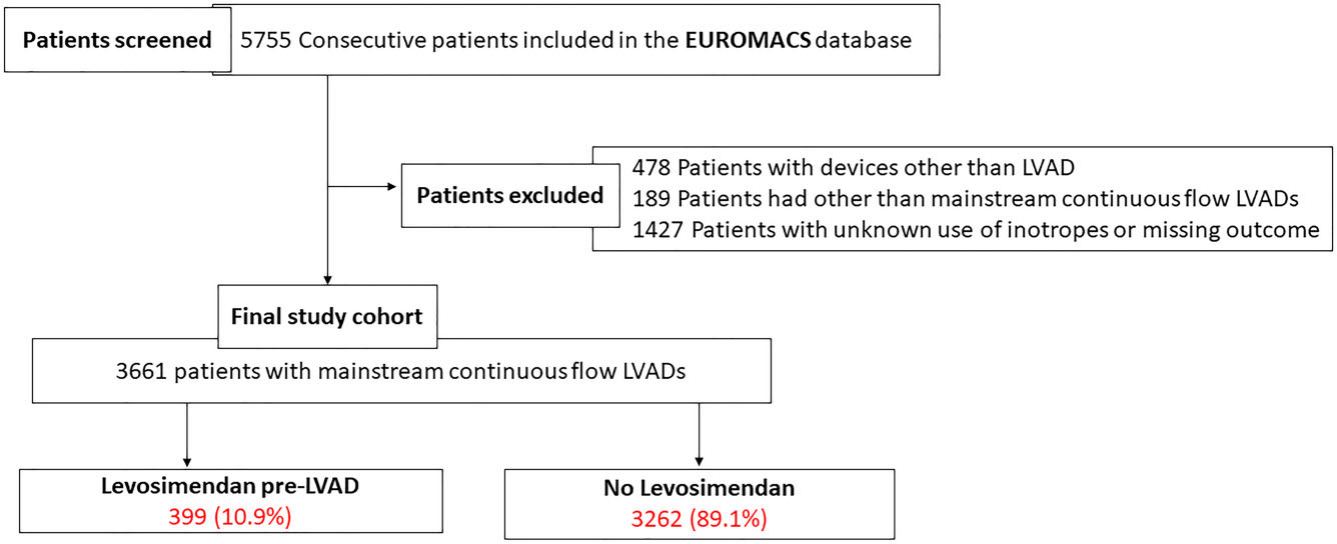
Flow chart.

All patients operated on between 1995 and 2021 were identified. We excluded patients with primary devices other than an LVAD (such as a total artificial heart and single right ventricle assist devices) (*n* = 478). Devices other than mainstream, continuous flow pumps (HeartMate 2, HeartMate 3 and HeartWare) (*n* = 189) were also excluded (Fig. 1). The final study cohort comprised patients treated between 2006 and 2021.

### Study outcome

The primary outcome was the rate of RHF early post-LVAD implants, defined as the need for an RVAD implant or the postoperative need for continuous inotropic support for ≥14 days [14]. Secondary outcomes were (i) the need for an RVAD implant; (ii) the length of stay in the ICU post-LVAD implant and (iii) 30-day and (iv) 1-year deaths.

### Missing values

Multiple imputation by chained equations using the mice package in R was used to impute missing values. For multiple imputations, we included all variables from our analysis model, including outcomes, and also a group of exogenous variables that had less than 10% missingness to help impute missing values (more details in the Supplementary Material, Text S1).

### Statistical analyses

Continuous data are presented as mean (SD: standard deviation) (Gaussian distribution) or median [interquartile range (IQR)] (non-Gaussian distribution). Categorical data are presented as frequencies (percentage). Comparisons among continuous variables were made with the Student *t*-test or the Mann–Whitney test, as appropriate. Continuous data outside 3 standard deviations were considered erroneous and removed. This outlier removal process was applied to the raw data for variables that were symmetric, whereas those skewed variables were first log-transformed. To investigate the association between levosimendan and the occurrence of post-LVAD RHF or the need for RVAD insertion, we applied binary logistic regression, with the odds ratio of levosimendan as the key parameter of interest. For mortality, we used Cox regression, with the hazard ratio of levosimendan as the key parameter of interest. Finally, for the duration of the ICU stay, we used a linear regression model. For each outcome, we carried out a propensity score matched analysis within multiple imputations as follows for every patient using levosimendan: We used propensity score matching to find 2 controls in each imputed data set using a caliper width of 0.05. We tested the robustness of our results by allowing combinations of calipers 0.01 and 0.1 and ratios of 1:1. In the primary analyses, we first matched the individuals based on their probability of receiving levosimendan. We ran a logistic regression model of levosimendan on all covariates, then used the predicted probabilities from this model to match individuals, with each subject who was prescribed levosimendan matched with 2 non-levosimendan controls who had a similar probability of treatment. To account for missing data in the covariates, we imputed 20 complete data sets via chained equations and performed a propensity score matched analysis within each of the 20 imputed data sets. Because each imputed data set differs randomly, the number of matched controls differs from imputation to imputation. Although outcome data were used to impute covariates, no missing outcome data were imputed. We then analysed these matched data in a logistic, Cox or linear model, depending on the outcome, and combined these results using Rubin’s rules. To account for clustering within and between matched pairs, we used logistic and linear random effects models, allowing a random intercept for pairs and similarly robust Cox regression accounting for pairs.

We report the odds ratio (for early RHF and RVAD implants), hazard ratio (mortality) or regression coefficient (ICU stay) of levosimendan, with a corresponding 95% confidence interval (CI) and *P*-value. All analyses were done in R v4.1 [R Core Team (2021)] with the use of statistical packages mice and MatchThem.

### Levosimendan protocol

No standardized protocol for the administration of levosimendan among study sites was used. Furthermore, the use of levosimendan was dependent on its availability at the study sites. More details on the local levosimendan protocols are provided in the Supplementary Material, Text S2 and Table S1.

## RESULTS

**Figure 2:**
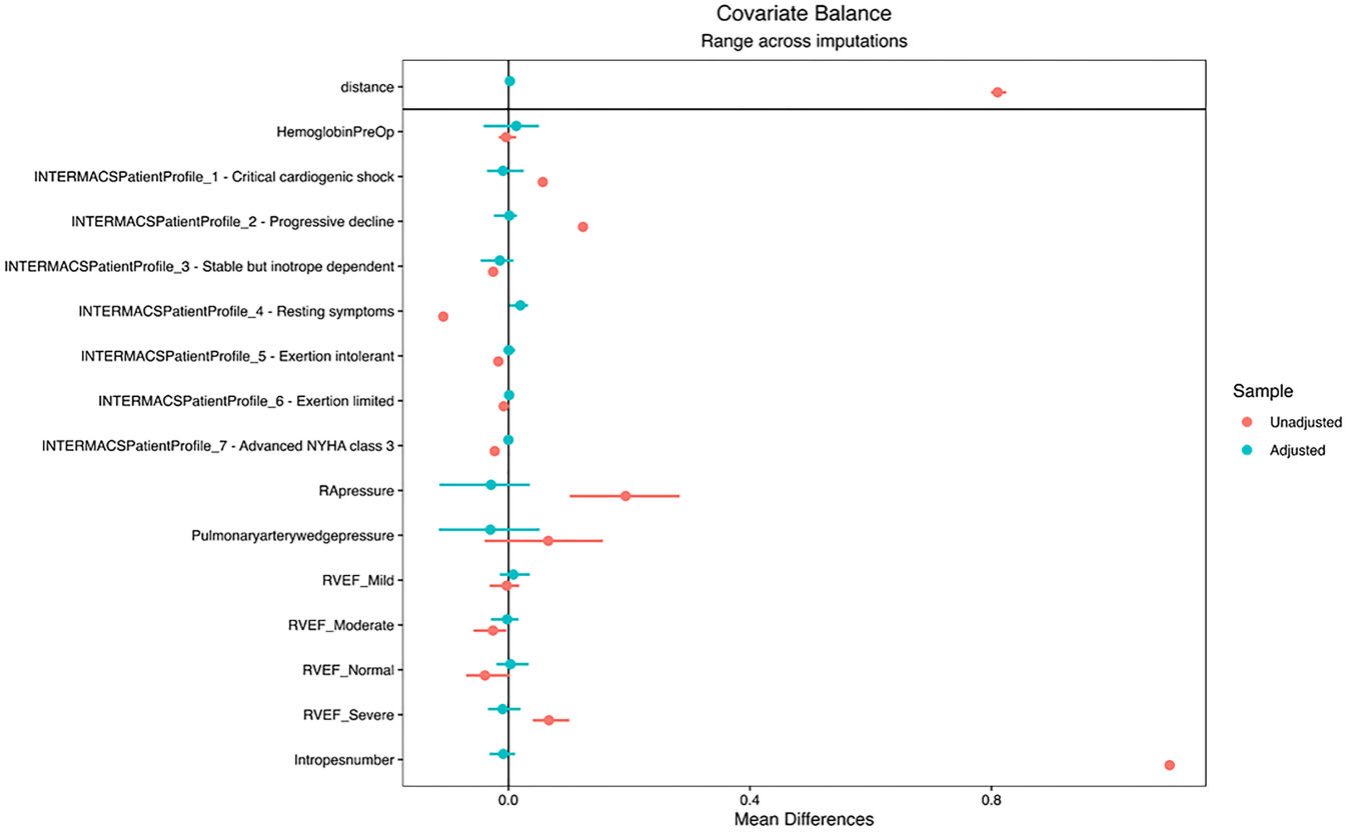
Love plot of covariate balance before and after matching.

In total, 3661 patients had a mainstream LVAD implant; 399 (10.9%) of these had received levosimendan before the LVAD was implanted. On average across the 20 imputations, we matched 357 patients receiving levosimendan to 622 control patients. Because matching is done within imputations, the number of patients receiving levosimendan and the controls matched each time vary. The balance before and after matching for each variable is shown in Fig. 2 and in Supplementary Table S2.

### Patient characteristics

Patient characteristics are presented in Table 1. Patients who received levosimendan (index cohort) had significantly higher EUROMACS RHF risk scores [median (IQR) 4.0 (2.0, 5.5) vs 2.0 (2.0, 4.0); *P* < 0.001]. In addition, they were younger, had more frequent ventilator use and had more often use of an intra-aortic balloon pump and ECMO. Moreover, they had higher right atrial pressure, lower serum haemoglobin levels and higher laboratory markers of renal and liver function.

**Table 1:** Characteristics of patients with or without levosimendan in *unmatched* data

Characteristic	Levosimendan n = 399	No levosimendan n = 3262	*P*-value
Age, years	54 (43, 61)	57 (49, 64)	<0.001
Male sex	332 (83%)	2767 (85%)	0.4
BSA (m^2^)	1.98 (1.83- 2.12)	1.97(1.82- 2.13)	0.8
White	369 (98%)	2547 (89%)	<0.001
Aetiology			
Dilated non-ischaemic	224 (57%)	1545 (49%)	0.014
Ischaemic	165 (42%)	1563 (49%)	0.014
Time since first diagnosis ≥2 years	232 (61%)	2112 (68%)	<0.001
Destination therapy	51 (13%)	815 (25%)	
IABP	57 (14%)	289 (9.0%)	<0.001
Ventilator	84 (21%)	420 (13%)	<0.001
ECMO	70 (18%)	380 (12%)	<0.001
INTERMACS class			
1	85 (21%)	507 (16%)	
2	163 (41%)	927 (29%)	
3	105 (26%)	938 (29%)	
4	37 (9.3%)	650 (20%)	
5	8 (2.0%)	119 (3.7%)	
6	1 (0.3%)	33 (1.0%)	
7	0 (0%)	72 (2.2%)	
Moderate/severe peripheral oedema	132 (40%)	910 (32%)	0.032
Loop diuretics	312 (81%)	2582 (82%)	0.074
Ascites	35 (13%)	236 (10%)	0.1
Use of ≥3 inotropes	189 (47%)	349 (11%)	
**ECG rhythm**			0.008
Sinus	184 (49%)	1570 (51%)	
Atrial fibrillation	52 (14.3%)	587 (18.6%)	
Paced	127 (34%)	878 (28%)	
Other	12 (3.2%)	48 (1.6%)	
**Echocardiographic results**			
LVEF <20%	235 (70%)	1643 (62%)	0.014
Severe mitral regurgitation	105 (30%)	572 (20%)	<0.001
Severe aortic regurgitation	2 (0.6%)	21 (0.8%)	0.014
Tricuspid regurgitation			0.015
None	22 (6.3%)	262 (9.4%)	
Trivial	40 (11%)	451 (16%)	
Mild	121 (35%)	957 (34%)	
Moderate	109 (31%)	769 (27%)	
Severe	58 (17%)	360 (13%)	
RVF			0.2
Normal	42 (17%)	463 (22%)	
Mild	67 (28%)	567 (27%)	
Moderate	91 (38%)	824 (39%)	
Severe	41 (17%)	281 (13%)	
TAPSE (mm)	15 (12- 18)	15 (12- 17)	0.9
**Haemodynamic values**			
Systolic PAP (mmHg)	50 (40- 62)	50 (39- 63)	0.7
RA pressure (mmHg)	12.0 (8- 17)	11 (7-15)	<0.001
PCWP (mmHg)	25 (19- 31)	24 (18- 30)	0.086
SVR	1288(917-1817)	1333(1003-1768)	0.5
PVR	220 (156- 348)	226 (144- 358)	>0.9
**Laboratory values (pre-LVAD)**			
Creatinine (mg/dl)	123 (91- 185)	114 (87- 154)	<0.001
AST (U/l)	40 (25- 81)	32 (22- 58)	<0.001
Total bilirubin (mg/dl)	1.45 (0.90- 2.30)	1.20(0.75- 2)	<0.001
Albumin (g/dl)	514 (420- 594)	503 (420- 580)	0.048
Haemoglobin (g/dl)	10.9(9.6-12.97)	11.7(10-13.5)	<0.001

ALT: aspartate aminotransferase; ECMO: extracorporeal membrane oxygenation; ECG: electrocardiogram; IABP: intra-aortic balloon pump; INTERMACS: Interagency Registry for Mechanically Assisted Circulatory Support; LVAD: left ventricular assist device; LVEF: left ventricular ejection fraction; PAP: pulmonary atrial pressure; PCWP: pulmonary capillary wedge pressure; PVR: pulmonary vascular resistance; RA: right atrium; RVF: right ventricle function; SVR: systemic vascular resistance; TAPSE: tricuspid annular plane systolic excursion.

Patients who received levosimendan had a significantly greater need for an RVAD implant [32 (8.0%) vs 178 (5.5%); *P* = 0.038], although there was no significant difference regarding the occurrence of post-LVAD early severe RHF [94 (24%) vs 680 (21%); *P* = 0.2] (Table 2).

**Table 2:** Outcomes of patients with or without levosimendan in *unmatched* data

Characteristic	Levosimendan, n = 399	No levosimendan n = 3262	*P*-value
RHF EUROMACS risk score	4 (2-5.5)	2 (2- 4)	<0.001
RHF	94 (24%)	680 (21%)	0.2
Need for RVAD insertion			0.038
No RVAD	367 (92%)	3,084 (95%)	
RVAD	32 (8.0%)	178 (5.5%)	
30-Day mortality	44 (11%)	364 (11%)	>0.9
1-Year mortality	112 (28%)	889 (27%)	0.7
ICU stay duration post-LVAD implant	19 (8- 35)	11 (5- 25)	<0.001

ICU: intensive care unit; LVAD: left ventricular assist device; RHF: right-sided heart failure; RVAD: right ventricular assist device.

Patients who received levosimendan had more days in the ICU [median (IQR) 19 (8, 35) vs 11 (5, 25); *P* < 0.001] (Table 2).

There was no significant difference between the 2 cohorts regarding the post-LVAD 30-day [44 (11%) vs 364 (11%); *P* > 0.9] and 1-year [112 (28%) vs 889 (27%); *P* = 0.7] mortality (Table 2).

### Post-left ventricular assist device right-sided heart failure and the need for a right ventricular assist device implant

Using propensity score matching, the logistic regression model showed no evidence for an association between levosimendan and post-LVAD early severe RHF [odds ratio 1.32, 95% CI 0.91, 1.84; *P* = 0.14] or the need for an RVAD implant [odds ratio 1.60, 95% CI 0.86, 3.00; *P* = 0.14] (Table 3).

**Table 3:** Logistic, Cox and linear models of different outcomes of patients taking levosimendan in *propensity score matched* pairs pooled across 20 imputed data sets, using robust methods to account for correlation within pairs

Variable	Outcome	OR/HR/coefficient	Lower 95% confidence interval	Upper 95% confidence interval	*P*-value
Levosimendan	RHF^a^	1.32	0.91	1.84	0.14
	Need for RVAD^a^	1.60	0.86	3.00	0.14
	Duration of ICU stay^b^	4.48	−1.66	10.62	0.14
	30-Day mortality^c^	0.83	0.52	1.29	0.39
	One-year mortality^c^	0.78	0.59	1.06	0.13

Regression type:

a Logistic mixed effect model.

b Linear mixed effect model.

c Robust Cox regression.

HR: hazard ratio; ICU: intensive care unit; OR: odds ratio; RHF: right-sided heart failure; RVAD: right ventricular assist device.

### Length of stay in the intensive care unit post-left ventricular assist device implant

Using propensity score matching across multiple imputations, the pooled linear mixed effect model showed no evidence of an association between the pre-LVAD use of levosimendan and the post-LVAD length of stay in the ICU (4.48 days longer on average in the levosimendan group, 95% CI 1.66, 10.62 days; *P* = 0.14) (Table 3).

### 30-Day and 1-year mortality

**Figure 3:**
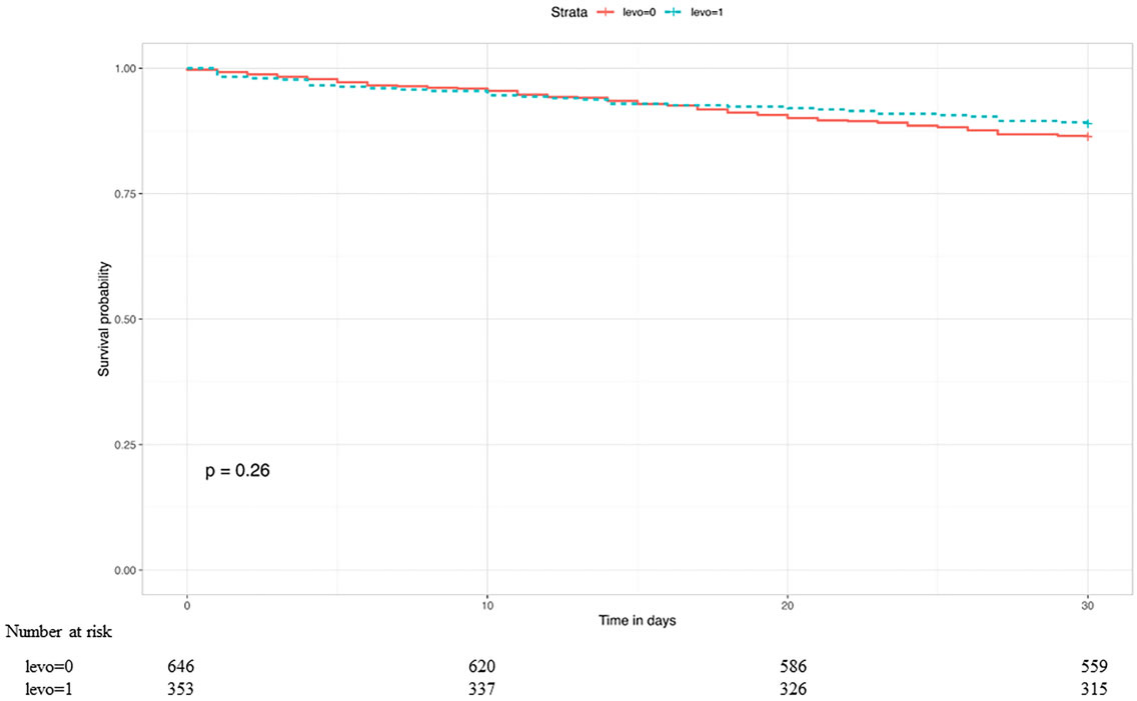
Kaplan–Meier curve showing the probability of 30-day survival in the levosimendan group (blue) versus that in a matched no-levosimendan group (red). This plot shows 1 of 20 matched imputed data sets. levo=Levosemindan.

**Figure 4:**
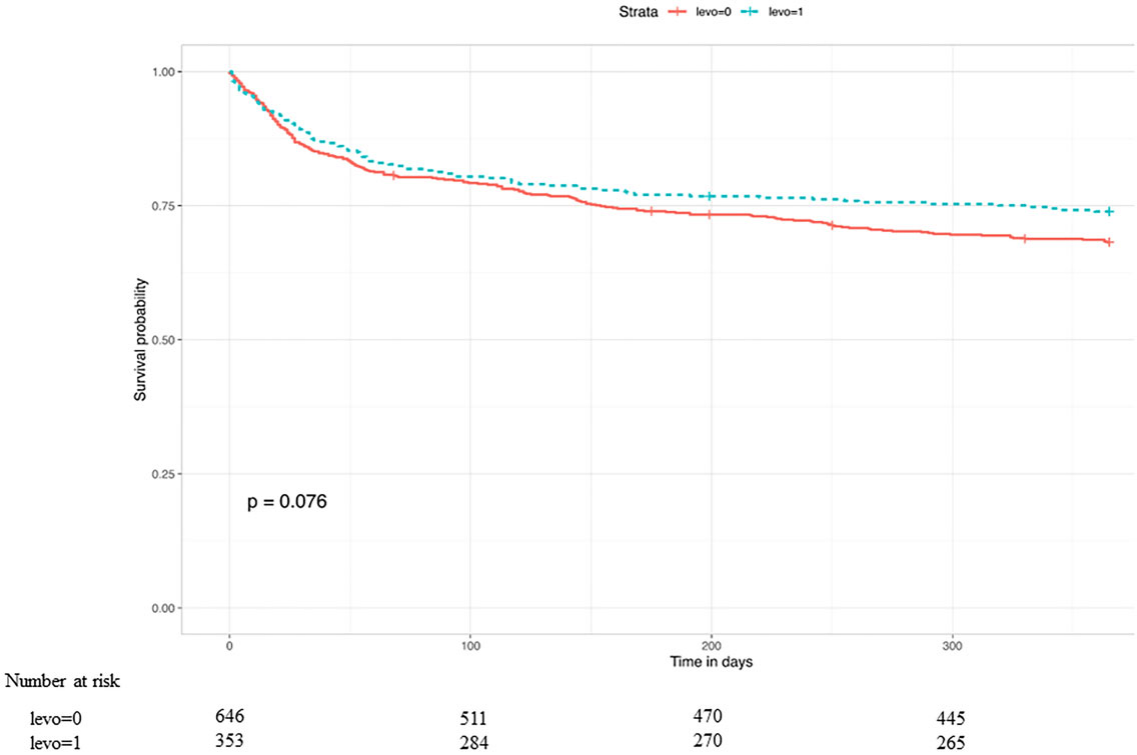
Kaplan–Meier curve showing the probability of 1-year survival in the levosimendan group (dotted curve) versus that is a matched no-levosimendan group (continuous curve). This plot shows 1 of 20 matched imputed data sets.

In our propensity score matched Cox regression model, there was no evidence of an association between the pre-LVAD use of levosimendan and the post-LVAD 30-day [hazard ratio (HR) 0.83, 95% CI 0.52, 1.29; *P* = 0.39] or 1-year mortality (HR 0.78, 95% CI 0.59, 1.06; *P* = 0.13) (Table 3). Kaplan–Meier curves of the survival probability over 30 days and 1 year are shown in Figs. 3 and 4 with log-rank test *P*-values of 0.26 and 0.076, respectively.

### Outcomes of different groups according to the European Registry for Patients with Mechanical Circulatory Support right-sided heart failure risk score

We compared the study outcomes within the different groups (high-, medium- and low-RHF risk) according to the EUROMACS RHF risk score between the 2 cohorts. There was no significant difference regarding the primary or secondary outcomes between the 2 cohorts in the comparisons of the different groups apart from the longer stay in the ICU of patients who received levosimendan in the high-risk group [median (IQR) 20 (8, 36) vs 14 (6, 29); *P* = 0.039] (Supplementary Material, Tables S6–S8).

### Testing the robustness of propensity score matching

We repeated the logistic, Cox and linear models of the 5 different outcomes of patients on levosimendan while varying the propensity score mechanism to test robustness across callipers and the number of matched controls. The primary analysis used 1:2 matching, i.e. 2 controls for every patient taking levosimendan, with a calliper width of 0.05. Supplementary Table S3 shows that the results are robust when using a caliper of 0.01 when matching and a calliper of 0.1 when using 1:2 matching.Supplementary Table S4 shows that the results are robust when the caliper width is increased to 0.1, and Supplementary Table S5 shows that the results are robust when reducing the number of controls and using a 1:1 matching process.

## DISCUSSION

To the best of our knowledge, this is the largest study to evaluate the outcomes of the preoperative use of levosimendan in patients having LVAD implants. In the largest European LVAD registry, we found that patients who received levosimendan had a significantly higher risk of developing RHF (based on the EUROMACS RHF score), received more RVADs in the early postoperative period and stayed longer in the ICU. However, there was no significant difference regarding the occurrence of post-LVAD early severe RHF or death (at 30 days and 1 year). Moreover, in a propensity score matched cohort, we found no evidence of an association between the preoperative use of levosimendan and the occurrence of post-LVAD RHF, the need for an RVAD implant in the early postoperative period, the length of stay in the ICU, or death (at 30 days and at 1 year) following an LVAD implant. Although there was a trend towards a survival benefit at 1 year in the patients who received levosimendan (*P* = 0.076), we do not advise interpreting this to mean it would become ‘statistically significant’ with more data [15–17].

Early RHF is considered by many as the “Achilles’ heel” of LVAD therapy in terms of excess morbidity and mortality, based on data from the Interagency Registry for Mechanically Assisted Circulatory Support and the EUROMACS registries [6, 12, 18–20]. Yet, there is no standardized protocol for the management and mitigation of early post-LVAD severe RHF. Typically, prolonged IV infusion of conventional inotropes is used to support patients who developed signs of RHF post-LVAD. Likewise, conventional inotropic support is used pre LVAD to optimize patients before surgery. Unfortunately, excess and prolonged use of these conventional inotropes is associated with poor outcomes [10]. In contrast, levosimendan is a relatively novel inotropic agent with unique characteristics. It is a first-in-class inotropic agent that acts as a calcium sensitizer. Moreover, it increases cardiac output and stroke volume and reduces peripheral vascular resistance without an increased risk of arrhythmia or myocardial “exhaustion”. In addition, levosimendan has a long therapeutic effect that may last for weeks [7].

In a recently published meta-analysis from our group, levosimendan use in patients undergoing ECMO was associated with significant veno-arterial ECMO weaning success and a lower risk of death [9]. In addition, another meta-analysis by our group demonstrated that levosimendan use in ambulatory patients with refractory HF has been associated with a wide range of improved haemodynamics, improved echocardiographic parameters, reverse left ventricular remodelling, lower filling pressures and lower levels of biomarkers of LV failure compared to those who did not receive levosimendan [10]. More recently, Yalcin *et al.*[21] reported the successful use of intermittent levosimendan infusion for the treatment of a patient with late RHF post-LVAD. In another meta-analysis about the perioperative use of levosimendan in patients undergoing cardiac surgery, the authors concluded that levosimendan improved the survival and cardiac output and reduced the number of cases of acute kidney injury and the need for renal replacement therapy in the postoperative period [22].

Data about using levosimendan pre-LVAD implant is limited [11]. In a recently published systematic review [11], only 2 studies met the predefined inclusion criteria [23, 24]. The 2 studies showed that there are at least haemodynamic improvements alongside improved organ perfusion associated with the use of levosimendan in patients undergoing LVAD. However, no survival benefits have been shown for the pre-LVAD use of levosimendan infusion, probably due to the relatively small sample. In contrast to these 2 small studies, our cohort included a larger number of patients and had a matched group of patients who did not receive levosimendan. In our study, most patients who received levosimendan had higher EUROMACS RHF scores and therefore had a high probability of post-LVAD RHF. Most of the centres had used levosimendan as an add-on medication in patients at high risk for RHF or even early signs of RV dysfunction or RHF before LVAD surgery. The fact that, despite a higher EUROMACS RHF risk score at baseline, the patients had similar outcomes might suggest a possible benefit of levosimendan therapy if a proper randomization experiment is conducted in such a population. Nevertheless, regression modelling after propensity score matching showed that there is no evidence for an association between levosimendan and better outcomes.

Importantly, the pathophysiology of post-LVAD RHF is complex and is not well described [25, 26]. It is not only due to right ventricular pump dysfunction, but it is a multifactorial mechanism where some factors cannot be corrected by levosimendan, especially in patients with poor RV function and poor RV reserve.

We did not investigate the safety of using levosimendan pre LVAD because our database is not designed to specifically address the use of levosimendan. However, authors of a previous studies including a meta-analysis reported that the administration of levosimendan was safe and well-tolerated without excess signs of arrythmia, tachycardia or hypotension compared to placebo [9, 10].

Based on the promising results of levosimendan in ambulatory patients with advanced HF, in patients undergoing cardiac surgery, in patients undergoing ECMO and the equipoise benefits of levosimendan in this study, we suggest initiating a large-scale randomized clinical trial to ascertain the clinical benefits of using levosimendan in patients taking LVADs.

## LIMITATIONS

We acknowledge important limitations to our study. First, this is a retrospective analysis, and the large multicentre databases are not designed for specific questions like the preoperative use of levosimendan in patients on LVADs, detailed criteria for preoperative RV failure and indications for and the timing of RVAD implants in individual centres. Therefore, other potentially relevant outcomes such as the haemodynamic and laboratory effects of levosimendan were not investigated. Second, as in most large-scale multicentre, multinational registries, data were missing: Although most variables of interest had below 30% missing values, we accepted only up to 10% missing values in exogenous variables used for multiple imputation. On the other hand, the EUROMACS database collects many variables, making it more likely that missing data could be predicted from the other observed variables, thereby strengthening the missing-at-random assumption. In addition, since last year, the EUROMACS investigators intensified their quality control measures to improve completeness of the data in the future. Third, data of patients treated before 2011 in the EUROMACS registry were collected retrospectively; therefore, it could be suboptimal. Fourth, the levosimendan infusion protocol differed among the EUROMACS participating centres and protocols within centres changed during the period of the study. However, our analysis showed that levosimendan used in the European centres was given to high-risk patients.

## CONCLUSIONS

We evaluated the outcome of using levosimendan infusion pre-LVAD implant in the EUROMACS population. In the unmatched cohort, the patients who received levosimendan pre-LVAD implant had a greater need for mechanical RV support with longer time in the ICU but similar RHF and death rates despite higher EUROMACS RHF scores at baseline in comparison to the patients who did not receive levosimendan. In matched patients, we found no evidence of an association between the use of levosimendan pre-LAVD implant and RHF or death. Further investigation with an adequately powered multicentre, randomized placebo-control study is warranted.

## Supplementary Material

ezad095_Supplementary_Data

## Data Availability

All relevant data are within the manuscript and its supporting information files.

## Abbreviations

ECMO: extracorporeal membrane oxygenation
EUROMACS: European Registry for Patients with Mechanical Circulatory Support
HF: heart failure
LVAD: left ventricular assist device
RHF: right-sided heart failure
RVAD: right ventricular assist device
