# Supplementary material for: Efficacy of levosimendan infusion in patients undergoing a left ventricular assist device implant in a propensity score matched analysis of the EUROMACS registry—the Euro LEVO-LVAD study
Source: Eur J Cardiothorac Surg. 2023 Mar 13;63(5):ezad095. doi: 10.1093/ejcts/ezad095 (PMC10693438; doi:10.1093/ejcts/ezad095)
Supplement: ezad095_Supplementary_Data [file ezad095_supplementary_data.docx]

### Supplementary material

**Efficacy of Levosimendan Infusion in Patients Undergoing Left Ventricular Assist Device Implantation in a propensity score-matched analysis of the EUROMACS Registry – the Euro LEVO-LVAD Study**

Authors and affiliations:

Mahmoud Abdelshafy, MD^1,2,3^; Kadir Caliskan, MD, PhD^4^, Andrew J. Simpkin, PhD^5,6^; Ahmed Elkoumy, MD^1,2,7^; Jesse R. Kimman, MD^4,8^; Hagar Elsherbini, BSc^4^; Hesham Elzomor, MD^1,2,7^; Theo M.M.H. de By, MBA, PhD^9^; Can Gollmann-Tepeköylü MD, PhD^10^;

Michael Berchtold-Herz MD^11^; Antonio Loforte MD, PhD^12^; David Reineke MD^13^;

Felix Schoenrath, MD,PhD^14,15^; Lech Paluszkiewicz, MD, PhD^16^; Jan Gummert, MD, PhD^16^; Paul Mohacsi, MD, eMBA^17,18^; Bart Meyns, MD, PhD^19^; and Osama Soliman, MD, PhD^1,2,20^

1. Discipline of Cardiology, Saolta Healthcare Group, Galway University Hospital, Health Service Executive, Galway, Ireland
2. CORRIB Core Lab, University of Galway, Galway, Ireland.
3. Department of Cardiology, Al-Azhar University, Cairo, Egypt
4. Department of Cardiology, Erasmus MC University Medical Center, Rotterdam, the Netherlands.
5. School of Mathematical and Statistical Sciences, University of Galway, Galway, Ireland.
6. Insight Centre for Data Analytics, University of Galway, Galway, Ireland.
7. Islamic Center of Cardiology and Cardiac Surgery, Al-Azhar University, Cairo, Egypt.
8. Department of Intensive Care, Erasmus MC University Medical Center, Rotterdam, the Netherlands.
9. EACTS House, Windsor, United Kingdom.
10. Department of Cardiac Surgery, Medical University of Innsbruck, Innsbruck, Austria.
11. Department of Cardiovascular Surgery, Faculty of Medicine, Heart Center Freiburg University, University of Freiburg, Freiburg, Germany.
12. Division of Cardiac Surgery, S. Orsola University Hospital, ALMA Mater Studiorum University of Bologna, IRCCS Bologna, Bologna, Italy.
13. Department of Cardiovascular Surgery, University Hospital, Berne, Switzerland.
14. Department of Cardiothoracic and Vascular Surgery, German Heart Center Berlin, Berlin, Germany.
15. DZHK (German Centre for Cardiovascular Research), Partner Site, Berlin, Germany.
16. Department for Thoracic and Cardiovascular Surgery, Heart and Diabetes Centre NRW, Ruhr-University Bochum, Bad Oeynhausen, Germany.
17. HerzGefässZentrum im Park, Zürich, Switzerland.
18. Department of Internal Medicine, Division of Cardiology, Medical University of Graz, Graz, Austria.
19. Katholieke Universiteit Leuven, Leuven, Belgium.
20. CÚRAM Centre for Medical Devices, Galway, Ireland.

**Supplementary Text 1**

**Missing values and multiple imputation**

For multiple imputation, we include a group of exogenous variables which have less than 10% missingness to help impute missing values in those modelled variables listed below. These include:

- Blood Type (A, AB, B, O),
- Age (Years),
- Gender (Male/Female),
- Mitral Regurgitation (None, Trivial, Mild, Moderate, Severe),
- Tricuspid Regurgitation (None, Trivial, Mild, Moderate, Severe),
- Aortic Regurgitation (None, Trivial, Mild, Moderate, Severe),
- Ejection Fraction Grade (<19%, 20-29%, 30-39%, 40-50%, >50%),
- Tricuspid Annular Plane Systolic Excursion (TAPSE, mm),
- Volume Status Peripheral Edema (None, Trivial, Mild, Moderate, Severe),
- Cardiac Rhythm (Atrial Fibrillation, Atrial Flutter, Paced, Sinus, Other),
- Body Surface Area (BSA, $m^{2}$),
- Pulmonary Artery Systolic Pressure (mmHg),
- Systemic Vascular Resistance (SVR, Dynes/Seconds/$cm^{-5}$),
- Pulmonary Vascular Resistance (PVR, Dynes/Seconds/$cm^{-5}$),
- Loop Diuretics (Never Used, Previously Used, Currently Used),
- Blood Urea Nitrogen (Mg/Dl, Pre-Surgery),
- SGPT ALT (Units/L, Pre-Surgery),
- SGOT AST (Units/L, Pre-Surgery),
- Total Bilirubin (Mg/Dl, Pre-Surgery),
- Albumin (G/L, Pre-Surgery),
- B-Type Natriuretic Peptide (BNP, pg/Ml, Pre-Surgery),
- NT-Pro BNP (pg/Ml, Pre-Surgery),
- White Blood Cells (WBC Per Ml, Pre-Surgery),
- Platelet (Per Micro-Liter),
- International Normalized Ratio (INR, Seconds),
- C-Reactive Protein (Mg/L),
- Time Since First Cardiac Diagnosis (<1 Month, 1-12 Months, 12-24 Months, >24 Months),
- Ethnic Origin (White/Non-White),
- Primary Diagnosis (Dilated Non-Ischemic, Ischemic, Restrictive),
- Cardiac Arrest (Yes/No),
- Dialysis (Yes/No),
- Intra-Aortic Balloon Pump (IABP, Yes/No),
- Ventilator (Yes/No),
- Extra-Corporeal Membrane Oxygenation (ECMO, Yes/No),
- Diabetes (Yes/No),
- Insulin Dependent (Yes/No),
- Chronic Obstructive Pulmonary Disease (COPD, Yes/No),
- current device strategy (Bridge to recovery, Bridge to transplant, Destination therapy, Possible bridge to transplant, Rescue therapy, Other),
- ascites (yes/no),
- creatinine (mg/dL, pre-surgery)
- Rhesus factor (positive/negative)
- Heart rate (beats per minute)
- Systolic BP (mmHg)
- Diastolic BP (mmHg)
- Height (cm)
- Weight (kg)
- ARB on admission (never, previous, current)
- Amiodarone on admission (never, previous, current)
- ACE inhibitors on admission (never, previous, current)
- Beta blockers (never, previous, current)
- Aldosterone antagonist (never, previous, current)
- Anticoagulant therapy drugs status (never, previous, current)
- Sodium (mEq/L, pre-surgery)
- Potassium (mmol/L, pre-surgery)
- Intubation (yes/no)
- Major MI (yes/no)
- Cardiac surgery (yes/no)
- Other surgical procedures (yes/no)
- Major infections (yes/no)
- Other VAD (yes/no)
- Ultrafiltration (yes/no)
- Feeding tube (yes/no)
- History of neurological event (None, CVA, ICB, TIA)
- Time in OR for implant (minutes)
- Device type (LVAD, LVAD/RVAD)
- Device brand LVAD (HeartMate II, HeartWare HVAD, Thoratec - HeartMate III)
- LVAD type of driving (Pulsating, Rotary)
- LVAD cannulae location in flow (LA appendage, LA interatrial groove, LV apex)
- LVAD cannulae location out flow (Ascending aorta, Descending thoracic aorta)
- Echo (yes/no)
- Swan Ganz (yes/no)
- Congestive heart failure assessment (yes/no)
- Body mass index ($kg/m^{2}$)
- Hospital stay (days)

**Supplementary Text 2**

**Levosimendan protocol**

Twenty-two centres were identified from the EUROMACS database using levosimendan and a questionnaire about the indication, timing, dose, and duration of levosimendan. Eleven centres (50%) respond to our questionnaire.

**Supplementary Table S1.** Levosimendan protocol among different centres

| **Centre** | **Indication** | **Timing** | **Dose** |
| --- | --- | --- | --- |
| 1 | All patients routinely | pre-LVAD | Bolus 12-24 mcg/kg in 10 minutes, then 0.1 mcg/kg/min in 24h. |
| 2 | NR | 2 days pre-LVAD | NR |
| 3 | Some patients with severely impaired RV and some without RV failure. | 10 days pre-LVAD, post-LVAD | No bolus. |
| 4 | Severely impaired RV. | pre-LVAD | 0.2 mcg/Kg/min for 24 hours or (0.1 mcg/Kg/min) for 48 hours, depending on the clinical situation. |
| 5 | NR | NR | NR |
| 6 | NR | NR | NR |
| 7 | Inodilator for poor RV. | pre-LVAD | No bolus dose, 0.1 or 0.05 mic/kg/min depending on the clinical situation. |
| 8 | No fixed protocol | pre-LVAD, post-LVAD | NR |
| 9 | NR | pre-LVAD, post-LVAD (rare) | 0.1 µg/kg/min for 24 hours |
| 10 | NR | NR | NR |
| 11 | NR | NR | NR |
| 12 | NR | NR |  |
| 13 | Routine in most elective patients. | 5 days pre-LVAD implantation. | 12.5mg over 24h approx. |
| 14 | NR | NR | NR |
| 15 | Patients with right heart failure. It was used for a short time and is no longer in our routine. | NR | NR |
| 16 | NR | NR | NR |
| 17 | NR | NR | NR |
| 18 | Patients who are listed high urgently, patients in IM 4 with impaired right heart function or have secondary organ failure but stable hemodynamics. | pre-LVAD | 0.1 mic/kg/min without loading, if systolic blood pressure is <100, start with 0.05. Duration 24-36h |
| 19 | NR | NR | NR |
| 20 | No fixed protocol | NR | NR |
| 21 | NR | pre-LAVD, post-LVAD | 0.1 mcg/kg/min |
| 22 | In semi-elective LVAD implantations | 2 days pre-LVAD | 0.1mcg/kg/min, going up to 0.2mcg/kg/min if tolerated well. |

NR= no response

**Supplementary Table S2.** Covariate balance before and after matching

| **Summary of Balance for All Data** | | | | | | |
| --- | --- | --- | --- | --- | --- | --- |
|  | Means Treated | Means Control | Std. Mean Diff. | Var. Ratio | eCDF Mean | eCDF Max |
| distance | 0.273 | 0.0889 | 0.8171 | 4.7336 | 0.3163 | 0.4659 |
| Hemoglobin pre-LVAD | 13.6142 | 13.4079 | 0.0121 | 1.4165 | 0.0354 | 0.1189 |
| INTERMACS class |  |  |  |  |  |  |
| Class 1 | 0.213 | 0.1557 | 0.1399 | . | 0.0573 | 0.0573 |
| Class 2 | 0.4085 | 0.2851 | 0.2511 | . | 0.1234 | 0.1234 |
| Class 3 | 0.2632 | 0.2882 | -0.0568 | . | 0.025 | 0.025 |
| Class 4 | 0.0927 | 0.2005 | -0.3715 | . | 0.1078 | 0.1078 |
| Class 5 | 0.0201 | 0.0374 | -0.1238 | . | 0.0174 | 0.0174 |
| Class 6 | 0.0025 | 0.0104 | -0.1583 | . | 0.0079 | 0.0079 |
| Class 7 | 0 | 0.0227 | -0.1612 | . | 0.0227 | 0.0227 |
| RA pressure | 13.6441 | 12.6082 | 0.1232 | 0.959 | 0.0226 | 0.0747 |
| Pulmonary artery wedge pressure | 25.9875 | 25.2361 | 0.0916 | 0.6165 | 0.0192 | 0.0465 |
| RVF |  |  |  |  |  |  |
| Normal | 0.1554 | 0.2256 | -0.1939 | . | 0.0702 | 0.0702 |
| Mild | 0.2632 | 0.2517 | 0.0261 | . | 0.0115 | 0.0115 |
| Moderate | 0.3584 | 0.3709 | -0.0262 | . | 0.0125 | 0.0125 |
| Severe | 0.2231 | 0.1517 | 0.1713 | . | 0.0713 | 0.0713 |
| Inotropes number | 2.5464 | 1.1441 | 1.0949 | 1.4333 | 0.2003 | 0.3731 |
|  |  |  |  |  |  |  |
| **Summary of Balance for Matched Data** | | | | | | |
|  | Means Treated | Means Control | Std. Mean Diff. | Var. Ratio | eCDF Mean | eCDF Max |
| distance | 0.2187 | 0.2181 | 0.0028 | 1.0117 | 0.0005 | 0.0113 |
| Hemoglobin pre-LVAD | 13.5959 | 13.722 | -0.0074 | 0.9599 | 0.0089 | 0.0354 |
| INTERMACS Class |  |  |  |  |  |  |
| Class 1 | 0.2068 | 0.2011 | 0.0138 | . | 0.0057 | 0.0057 |
| Class 2 | 0.3853 | 0.3881 | -0.0058 | . | 0.0028 | 0.0028 |
| Class 3 | 0.2805 | 0.2847 | -0.0096 | . | 0.0042 | 0.0042 |
| Class 4 | 0.102 | 0.1006 | 0.0049 | . | 0.0014 | 0.0014 |
| Class 5 | 0.0227 | 0.0212 | 0.0101 | . | 0.0014 | 0.0014 |
| Class 6 | 0.0028 | 0.0042 | -0.0283 | . | 0.0014 | 0.0014 |
| Class 7 | 0 | 0 | 0 | . | 0 | 0 |
| RA pressure | 13.4731 | 13.7762 | -0.036 | 0.8159 | 0.0079 | 0.0297 |
| Pulmonary artery wedge pressure | 26.2323 | 26.4618 | -0.028 | 0.3788 | 0.0147 | 0.0453 |
| RVF |  |  |  |  |  |  |
| Normal | 0.1615 | 0.1629 | -0.0039 | . | 0.0014 | 0.0014 |
| Mild | 0.255 | 0.2479 | 0.0161 | . | 0.0071 | 0.0071 |
| Moderate | 0.3626 | 0.3824 | -0.0414 | . | 0.0198 | 0.0198 |
| Severe | 0.221 | 0.2068 | 0.034 | . | 0.0142 | 0.0142 |
| Inotropes number | 2.2833 | 2.2734 | 0.0077 | 1.0351 | 0.0237 | 0.0779 |

**Supplementary Table S3.** Logistic, Cox and linear models of different outcomes on Levosimendan in *propensity score-matched* pairs pooled across 20 imputed datasets, using robust methods to account for correlation within pairs [reduced calliper 0.01]

| **Variable** | **Outcome** | **OR/HR/ Coefficient** | **lower .95** | **upper .95** | **p-value** |
| --- | --- | --- | --- | --- | --- |
| Levosimendan | RHF^1^ | 1.32 | 0.90 | 1.94 | 0.15 |
|  | Need for RVAD^1^ | 1.66 | 0.85 | 3.24 | 0.14 |
|  | Duration of ICU stay^2^ | 3.50 | -1.97 | 8.97 | 0.21 |
|  | 30 day mortality^3^ | 0.82 | 0.52 | 1.30 | 0.39 |
|  | One year mortality^3^ | 0.74 | 0.52 | 1.01 | 0.06 |

Regression type: 1: logistic mixed effect model, 2: linear mixed effect model; 3: robust Cox regression

Abbreviations: see table 2.

**Supplementary Table S4.** Logistic, Cox and linear models of different outcomes on Levosimendan in *propensity score-matched* pairs pooled across 20 imputed datasets, using robust methods to account for correlation within pairs [increased calliper 0.1]

| **Variable** | **Outcome** | **OR/HR/ Coefficient** | **lower .95** | **upper .95** | **p-value** |
| --- | --- | --- | --- | --- | --- |
| Levosimendan | RHF^1^ | 1.32 | 0.94 | 1.84 | 0.11 |
|  | Need for RVAD^1^ | 1.58 | 0.85 | 2.90 | 0.15 |
|  | Duration of ICU stay^2^ | 4.65 | -0.65 | 9.95 | 0.09 |
|  | 30 day mortality^3^ | 0.81 | 0.53 | 1.23 | 0.31 |
|  | One year mortality^3^ | 0.78 | 0.60 | 1.02 | 0.07 |

Regression type: 1: logistic mixed effect model, 2: linear mixed effect model; 3: robust Cox regression

Abbreviations: see table 2.

**Supplementary Table S5.** Logistic, Cox and linear models of different outcomes on Levosimendan in *propensity score-matched* pairs pooled across 20 imputed datasets, using robust methods to account for correlation within pairs [same calliper 0.01 but using 1:1 matching, i.e. fewer controls]

| **Variable** | **Outcome** | **OR/HR/ Coefficient** | **lower .95** | **upper .95** | **p-value** |
| --- | --- | --- | --- | --- | --- |
| Levosimendan | RHF^1^ | 1.34 | 0.88 | 2.06 | 0.18 |
|  | Need for RVAD^1^ | 2.33 | 0.52 | 10.22 | 0.26 |
|  | Duration of ICU stay^2^ | 4.64 | 02.53 | 11.80 | 0.20 |
|  | 30 day mortality^3^ | 0.83 | 0.51 | 1.38 | 0.47 |
|  | One year mortality^3^ | 0.79 | 0.57 | 1.07 | 0.13 |

Regression type: 1: logistic mixed effect model, 2: linear mixed effect model; 3: robust Cox regression

Abbreviations: see table 2.

**Supplementary Table S6.**  Outcomes of patients with or without Levosimendan in the high-risk group (RHF EUROMACS risk score over 4)

| **Characteristic** | **Levosimendan**  **n = 106** | **No Levosimendan**  **n = 472** | **p-value** |
| --- | --- | --- | --- |
| RHF | 25 (24%) | 122 (26%) | >0.9 |
| Need for RVAD insertion |  |  | >0.9 |
| - No RVAD | 99 (93%) | 435 (92%) |  |
| - RVAD | 7 (6.6%) | 37 (7.8%) |  |
| 30-days Mortality | 10 (9.4%) | 52 (11%) | >0.9 |
| 1-year Mortality | 31 (29%) | 135 (29%) | >0.9 |
| ICU stay duration | 20 (8- 36) | 14 (6- 29) | 0.039 |

Abbreviations, see Table 2.

**Supplementary Table S7.**  Outcomes of patients with or without Levosimendan in the medium-risk group (RHF EUROMACS risk score between 2.5 and 4)

| **Characteristic** | **Levosimendan**  **n = 16** | **No Levosimendan**  **n = 103** | **p-value** |
| --- | --- | --- | --- |
| RHF | 5 (31%) | 26 (25%) | >0.9 |
| Need for RVAD insertion |  |  | 0.6 |
| - No RVAD | 14 (88%) | 98 (95%) |  |
| - RVAD | 2 (12%) | 5 (4.9%) |  |
| 30-days Mortality | 2 (12%) | 9 (8.7%) | >0.9 |
| 1-year Mortality | 4 (25%) | 25 (24%) | >0.9 |
| ICU stay duration | 18 (6- 28) | 11 (4- 25) | >0.9 |

Abbreviations, see Table 2.

**Supplementary Table S8.** Outcomes of patients with or without Levosimendan in the low-risk group (RHF EUROMACS risk score below 2.5)

| **Characteristic** | **Levosimendan**  **n = 55** | **No Levosimendan**  **n = 780** | **p-value** |
| --- | --- | --- | --- |
| RHF | 7 (13%) | 99 (13%) | >0.9 |
| Need for RVAD insertion |  |  | >0.9 |
| - No RVAD | 53 (96%) | 754 (97%) |  |
| - RVAD | 2 (3.6%) | 26 (3.3%) |  |
| 30-days Mortality | 2 (3.6%) | 49 (6.3%) | >0.9 |
| 1-year Mortality | 3 (5.5%) | 125 (16%) | 0.105 |
| ICU stay duration | 8 (4- 15) | 7 (4- 14) | >0.9 |

Abbreviations, see Table 2.
